# Mice Exposed to Chronic Intermittent Hypoxia Simulate Clinical Features of Deficiency of both Qi and Yin Syndrome in Traditional Chinese Medicine

**DOI:** 10.1093/ecam/nep226

**Published:** 2011-06-08

**Authors:** Chengzhi Chai, Junping Kou, Danni Zhu, Yongqing Yan, Boyang Yu

**Affiliations:** Department of Complex Prescription of TCM, China Pharmaceutical University, Nanjing, Jiangsu 211198, China

## Abstract

Deficiency of both Qi and Yin Syndrome (DQYS) is one of the common syndromes in traditional Chinese medicine (TCM), mainly characterized by tiredness, emaciation, anorexia, fidget, palpitation and rapid pulse, and so forth. Currently, there is no available animal model which can reflect the clinical features of this syndrome. In the present paper, we observed the time-course changes of whole behavior, body weight, food intake, locomotive activity and electrocardiogram in mice exposed to chronic intermittent hypoxia for 6 weeks, and measured bleeding time at last according to the clinical features of DQYS and one key pathological factor. The results showed that the mice exposed to intermittent hypoxia for certain time presented lackluster hair, dull looking hair, resistance, attacking, body weight loss, food intake decline, locomotive activity decrease, heart rate quickening and T wave elevating, which were similar to the major clinical features of DQYS. Meanwhile, bleeding time shortening was also found, which was consistent with the clinical fact that DQYS often accompanied with blood stasis. The possible explanation was also outlined according to the available literature. Such findings suggested chronic intermittent hypoxia could induce similar symptoms and signs in mice accorded with the clinical features of DQYS, which provided a suitable animal model for evaluation of drugs for the treatment of this syndrome and further exploration of pathological process or correlation of the syndrome and related diseases.

## 1. Introduction

Traditional Chinese medicine (TCM) is one part of alternative medicine which has unique theoretic system, and has been demonstrated to be effective in the prevention and treatment of various diseases by its long history of practical clinical experience [1]. In the basic studies, animal models were often used to evaluate efficacy of Chinese drugs; however, those animal models aiming primarily at diseases only could not exactly reflect the characteristic of syndrome in TCM, due to the differences between theoretic systems of TCM and modern medicine.

Combination of differentiation syndrome and disease is the main therapeutic mode and feature of TCM [2]. Before modern medicine was introduced to China, practitioners of TCM diagnosed and treated diseases by symptoms and signs through inspection, listening and smelling, inquiring and palpation for thousands of years. Even today, despite modern methods of diagnosis develop rapidly, the methods mentioned above still play important role in the clinical practice of TCM. Studies have demonstrated that clinical findings obtained through conventional macroscopical methods often closely correlated with those from microscopical methods of modern medicine [3]. Therefore, we think that the first and most important step for constructing the animal model of syndromes of TCM is to reproduce the major symptoms and signs of syndromes based on possible common pathological factor of syndromes and related diseases.

DQYS is one of the common syndromes in TCM, characterized by tiredness, emaciation, anorexia, fidget, palpitation and rapid pulse, and so forth, which is often seen in the course of diseases such as cancer, cardiovascular diseases, respiratory diseases and chronic kidney diseases [4, 5]. Though a bunch of animal models have been established to simulate syndrome of traditional Chinese medicine, such as deficiency of Qi, deficiency of yin, blood stasis syndrome, and so forth, developed recently [6–8], and some scholars tried to construct DQYS animal model through deprivation the rats of rapid eye movement (REM) sleep for 96 h [9]. Till now, the suitable animal model accorded with the major clinical symptoms and signs of DQYS are still lacking.

It has been reported that oxidative stress is an important pathophysiologic process in the above-mentioned diseases related to DQYS [10–14]. Meanwhile, hypoxia often exists in these diseases and can definitely induce oxidative stress [15]. Studies have shown that mice exposed to chronic hypoxia showed the manifestations of body weight loss, food intake decline and sickness behavior [12, 16, 17], which were similar to some clinical features of DQYS; we then presumed that hypoxia might also be a key factor of this TCM syndrome. Therefore, in the present paper, we tried to verify whether mice exposed to chronic intermittent hypoxia can mimic main clinical symptoms and signs of DQYS.

## 2. Methods

### 2.1. Animals and Intermittent Hypoxia Treatment

Twenty specific pathogen-free ICR mice weighting 18–22 g were randomized into two groups: control group (*n* = 10) and model group (*n* = 10). Mice were housed in individual cages supplied with 15 g food per mouse daily. The animal handling procedures were in compliance with the China National Institutes of Healthy Guidelines for the Care and Use of Laboratory Animals and were approved by local committee review. The method of intermittent hypoxic was modified from acute hypoxia [18]. In brief, mice of the model group were placed into 200 mL of wide-mouthed bottle with 15 g soda lime for 20 min from 8 AM everyday. The oxygen concentration of the hypoxic chamber was measured by an oxygen meter (XT-668, China) at 1-min intervals and the mean oxygen concentration is 21.04% at min 1 and 6.83% at min 20. This 20-min cycle was repeated for 42 days. During the remaining time, the animals were maintained in normal environment.

### 2.2. Body Weight and Food Intake Measurement

Body weight and food intake of each mouse were monitored on days 1, 7, 14, 21, 28, 35 and 42 after exposure to hypoxia at 9:00 AM on a top-loading balance accurate to ±0.1 g.

### 2.3. Open-Field Test

The open-field test was performed on days 1, 7, 14, 21, 28, 35, and 42 according to the reported method [19]. The open field consisted of a circular arena (40 cm diameter, 38 cm high) with 19 quadrants drawn by concentric circles and lines. Animals were placed individually at the center of the arena and allowed to freely explore the new environment. The total ambulation and times of rearing were counted for 2 min.

### 2.4. Electrocardiogram Observation

Electrocardiogram was recorded on days 1, 14, 28 and 42 at 10 AM after intraperitoneal injection of 4% chloral hydrate (0.1 mL per 10 g). Heart rate and changes of T wave were calculated [20].

### 2.5. Bleeding Time Test

The tail transection bleeding time was determined according to the method of Dejana et al. [21]. At 9 AM on day 43 of experiment, the mouse tail was transected at 5 mm from the tip and then immediately immersed into warmed (37°C) saline. The bleeding time was recorded as latency from the tail transection to the moment the blood flow stopped for >60 s.

### 2.6. Statistical Analysis

The experimental results were expressed as the mean ± SEM. Repeated measures of ANOVA was carried out to test the statistical significance of body weight, food intake, open-field test and electrocardiogram results. Student's *t*-test was used to determine the difference of bleeding time and body weight, food intake, open-field test and electrocardiogram results at different observation points. All the analyses were conducted using the Statistical Package for the Social Sciences (SPSS) 13.0 for Windows (SPSS, Chicago, IL, USA). Differences were considered significant if the probability of error was <5%.

## 3. Results

### 3.1. Body Weight and Food Intake in Mice Exposed to Chronic Intermittent Hypoxia

**Figure 1:**
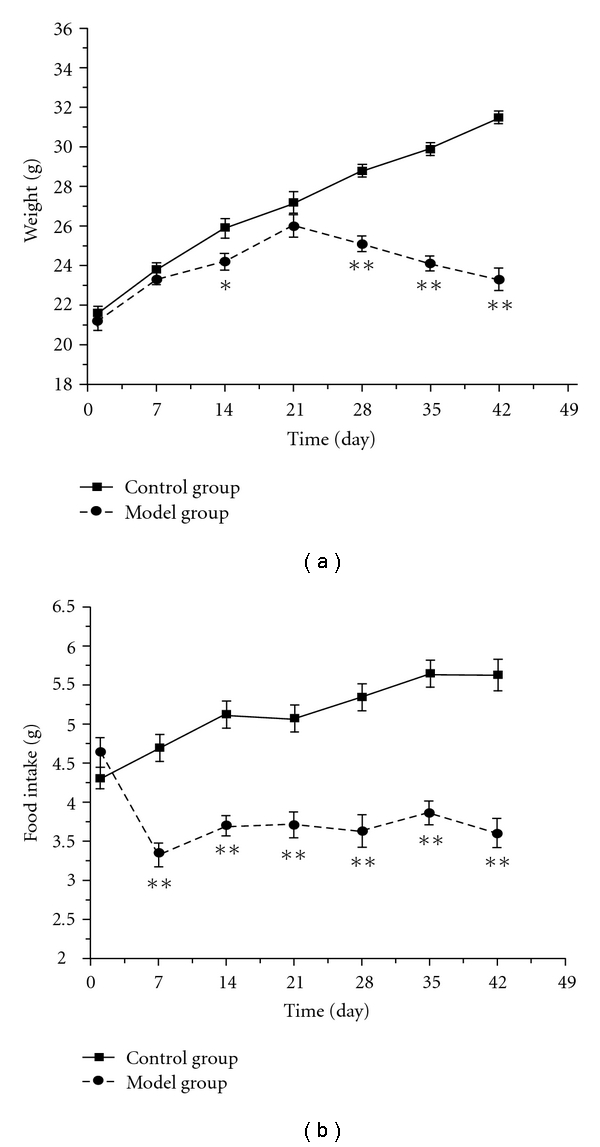
The time-course changes of body weight (a) and food intake (b) in mice exposed to intermittent hypoxia. Each value represents the mean ± SEM of 10 mice. Significance was evaluated with the *t*-test and repeated measures ANOVA. *P < .05, **P < .01, compared with the control group.

As shown in Figure 1(a), the body weight of the control group kept gaining during the experiment from 21.6 ± 0.3 g to 31.5 ± 0.3 g, while that of the model group gained slowly at early time, reached peak (26.0 ± 0.5 g) on day 21 and began to decrease significantly after being exposed to chronic intermittent hypoxia. On the other hand, the body weight and food intake basically altered in parallel. The food intake of the control group kept increasing gradually during the experiment from 4.3 ± 0.1 g to 5.6 ± 0.2 g, while that of the model group decreased remarkably from day 7 (3.3 ± 0.2 g) and maintained at the low level throughout the experiment (Figure 1(b)). Furthermore, all the mice of the model group manifest lackluster hair, dull looking hair, resistance, attacking, urinary frequency, and so forth. 

### 3.2. Open-Field Behavior in Mice Exposed to Chronic Intermittent Hypoxia

**Figure 2:**
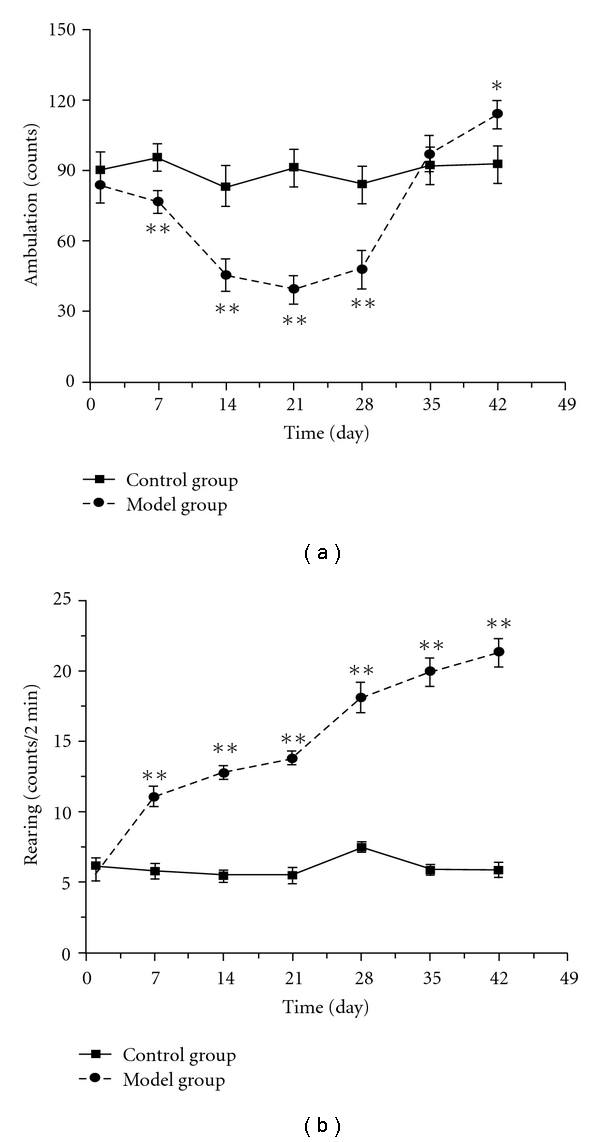
The time-course changes of ambulation (a) and rearing (b) counts in mice exposed to intermittent hypoxia. Each value represents the mean ± SEM of 10 mice. Significance was evaluated with the *t*-test and repeated measures ANOVA. *P < .05, **P < .01, compared with the control group.

The results in Figure 2 showed that the rearing and ambulation counts of the control group maintain at a steady level (90.3 ± 7.7 versus 92.5 ± 8.2 and 6.2 ± 0.6 versus 5.9 ± 0.5, on days 1 and 42 of experiment, resp.). The rearing counts of the model group increased significantly from days 7 to 42, while their ambulation counts declined markedly on day 7 and reached the lowest peak on day 21, but increased sharply to 114.0 ± 5.8 on day 42 after exposure to chronic intermittent hypoxia.

### 3.3. Electrocardiogram in Mice Exposed to Chronic Intermittent Hypoxia

**Figure 3:**
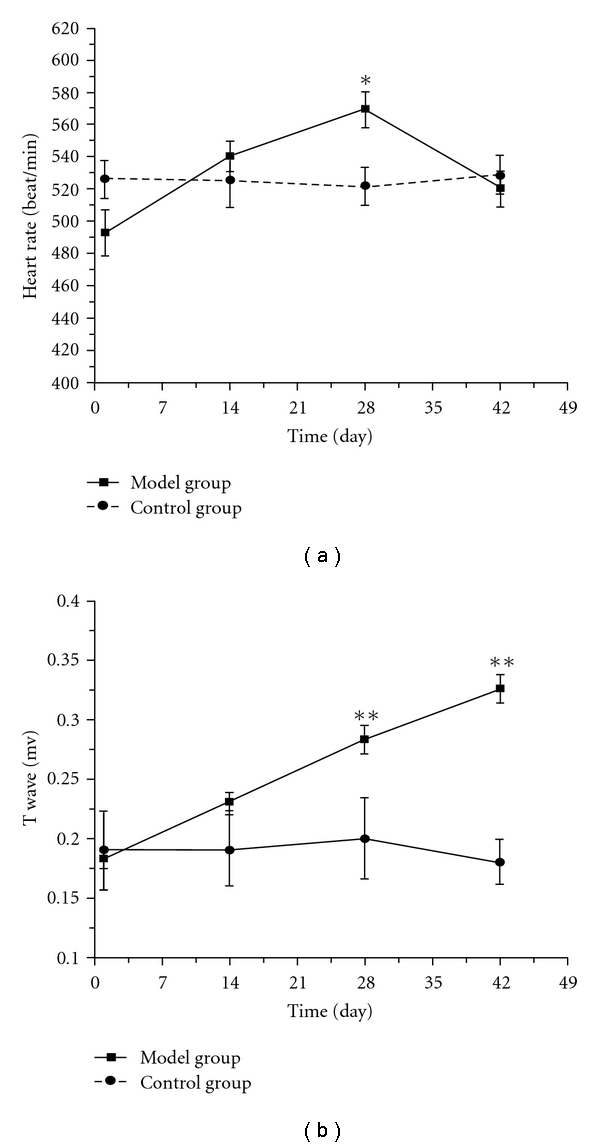
The time-course changes of heart rate (a), and T wave (b) in mice exposed to chronic intermittent hypoxia. Each value represents the mean ± SEM. Significance was evaluated with one-way repeated measures of ANOVA. *P < .05, **P < .01, compared with the self-control group.

**Figure 4:**
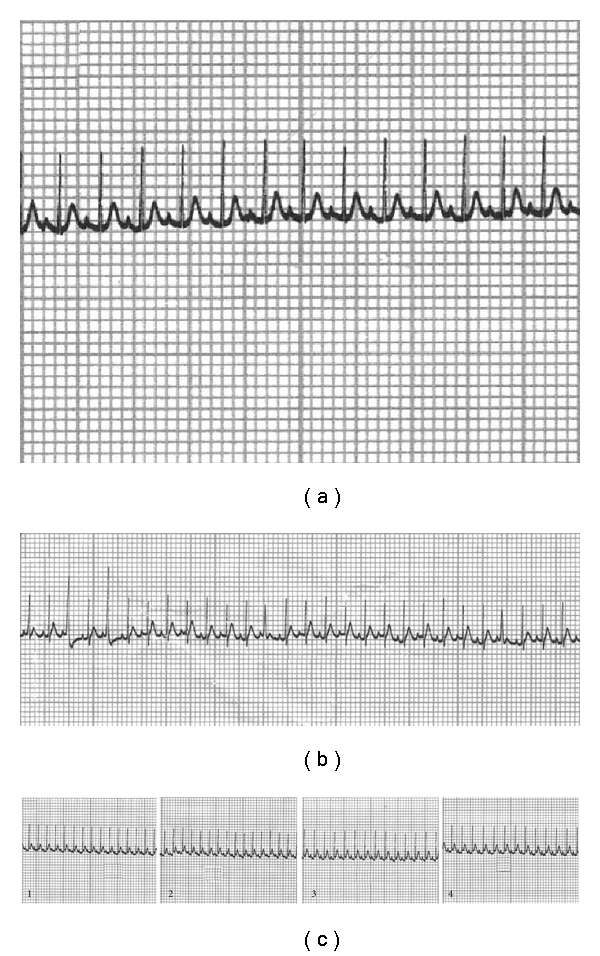
Normal electrocardiogram (a), premature beat (b), and T wave elevation (c) during experiment (1, 2, 3 and 4 were recorded on days 1, 14, 28 and 42 of experiment, resp.).

As shown in Figure 3(a), the heart rate of the control group maintained at a steady level throughout the experiment, while that of the model group increased from day 14, peaked on day 28, and then declined slightly on day 35 after exposure to chronic intermittent hypoxia. Meanwhile, the T wave of the model group increased gradually, and the elevation on days 28 and 42 was statistically significant from that on day 1, respectively (Figure 3(b)). During the experiment, six mice in the model group developed arrhythmia including premature beat and paroxysmal tachycardia, and their typical electrocardiogram were shown in Figure 4. 

### 3.4. The Change of Bleeding Time in Mice Exposed to Chronic Intermittent Hypoxia

**Figure 5:**
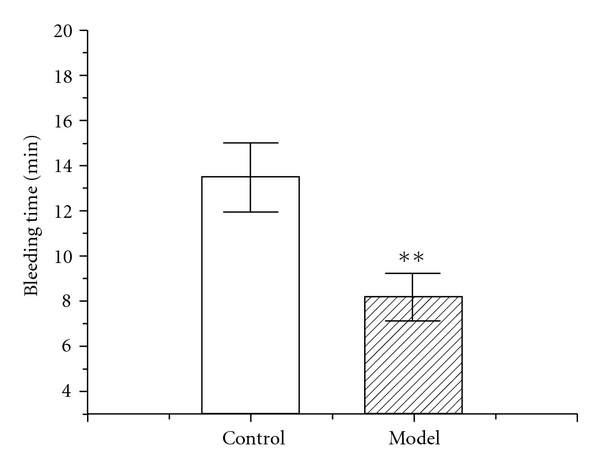
Change of bleeding time on Day 43 in mice exposed to chronic intermittent hypoxia. Each value represents the mean ± SEM of 10 mice. Significance was evaluated with one-way repeated measures of ANOVA. **P < .01, compared with the control group.

The results showed that the bleeding time of the model group decreased significantly after exposure to intermittent hypoxia for 42 days (Figure 5), compared to the control group (8.2 ± 3.3 min versus 13.5 ± 4.8 min).

## 4. Discussion

The whole manifestations of animal model should reflect the clinical features, which is the most important factor in constructing the animal model and is the qualification of further research. In the present paper, we observed the time-course changes of several whole manifestations in mice exposed to chronic intermittent hypoxia, according to the major symptoms and signs of DQYS and one common pathological factor of this syndrome and its related diseases. The results showed that mice exposed to intermittent hypoxia for certain time could simulate the symptoms and signs similar to the clinical features of this syndrome.

Firstly, we found that food intake of mice dropped significantly from day 7 and maintained at the low level, and the body weight of the model group gained slowly at early time, and decreased significantly after being exposed to intermittent hypoxia for 28 days (Figures 1(a) and 1(b)), which were similar to emaciation and anorexia in the clinical features of DQYS. Other studies also showed declination of body weight and food intake in mice exposed to chronic intermittent hypoxia [12, 16], which were consistent with our results.

Secondly, in the open-field test, we observed that the ambulation counts of the model group declined significantly at the early stage and then increased sharply at the late stage of the experiment (Figure 2(a)), while the rearing counts of the model group increased significantly from day 7 (Figure 2(b)), and counts of preening, defecation, urination, grooming and sniffing also enhanced (data not shown). The trend of ambulation counts was different from other study [17], which might be due to the different methods and observation points. It seemed that our findings were more similar to tiredness and fidget in the clinical symptoms of DQYS.

Furthermore, we also recorded electrocardiograms of mice every 2 weeks, considering that palpitation and rapid pulse were often seen in the clinical features of DQYS. Heart rate of the model group mice was found to increase gradually from day 14, peaked at day 28, and T wave elevation was significant from day 28 after intermittent hypoxia treatment. Such difference observed by self-comparison could exclude the bias of individual variability. Meanwhile, six mice in the model group developed some types of arrhythmia such as frequent premature beat, paroxysmal tachycardia, which were similar to palpitation and rapid pulse in the syndrome. Such results suggested that the manifestations of mice exposed to chronic hypoxia from days 14 to 35 were almost consistent with the clinical features of DQYS.

Finally, the shortened bleeding time indicating hypercoagulation status in the model group was found at the end of hypoxia treatment for 42 days, which not only accorded with the fact that blood stasis often existed in this syndrome but also accorded with the recent report that hypercoagulability connected with some related diseases [22–26].

On the other hand, many complex prescriptions are designed for some certain syndrome. As one of the typical complex prescriptions of DQYS, Shengmai-san (SMS) has been effectively used in the treatment of many diseases which are related to DQYS [27–29]. Our preliminary test showed that SMS could significantly improve the main signs such as body weight, food intake, T wave of electrocardiogram, and so forth, in mice exposed to chronic intermittent hypoxia, especially after being given orally for the first four weeks. Such results also provided some evidence about the relationship of this mouse model to DQYS.

**Figure 6:**
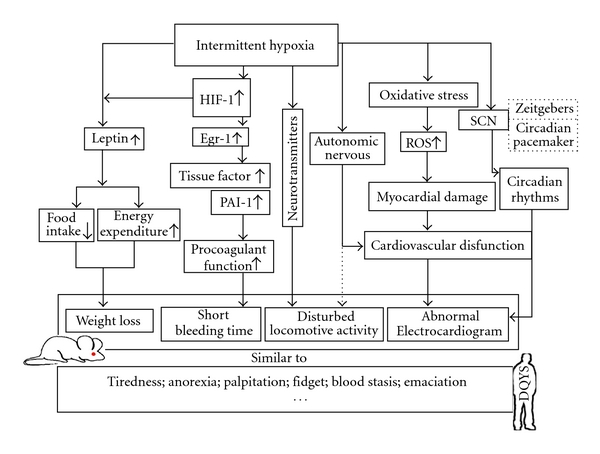
The probable mechanism attributed to the manifestations of mice exposed to intermittent hypoxia. HIF-1: hypoxia inducible factor-1; EGR-1: early growth response-1; PAI-1: plasminogen activator inhibitor-1; SCN: suprachiasmatic nucleus; ROS: reactive oxidative species.

Meanwhile, according to the theory of TCM, when some exterior signs appear, there must be some related interior changes. Our present data showed that mice exposed to chronic intermittent hypoxia displayed the similar signs to DQYS, which indicated some corresponding pathological changes associated with such exterior signs and could give some possible explanations for the formation of DQYS. Actually, many scholars have done plenty of work to explore the influence of hypoxia on physiological and biochemical regulatory systems. Although hypoxia induced variable results under different experimental conditions and with diverse animal species, some common pathological changes exist. It has been recorded that hypoxia could directly affect the output of the circadian pacemaker in the suprachiasmatic nucleus (SCN) or influence the response of the SCN to zeitgebers, which resulted in the disturbance of circadian rhythms [30–32]. Both sympathetic and parasympathetic nervous activity might have important roles in acclimatization to hypoxia. Disruptions of circadian rhythms often induced the changes of locomotive activity and abnormal cardiogram [33]. Injury to myocardium may be mediated by the generation of reactive oxygen species (ROS), and oxygen activated the transcription factor early growth response-1 (Egr-1) leads to *de novo* transcription/translation of tissue factor in mononuclear phagocytes and smooth muscle cells, which eventuates in vascular fibrin deposition. The procoagulant response is magnified by concomitant suppression of fibrinolysis by hypoxia-mediated upregulation of plasminogen activator inhibitor-1 [34]. Hypoxia also induced hypoxia-inducible factor 1 (HIF-1) expression so as to enhance the leptin secretion; leptin regulates food intake and energy expenditure and results in body weight loss [35–40]. Taken together, the series of events provided a possible context between the pathological mechanism of oxygen scarcity and the main clinical signs of DQYS, and we outlined the hypothetical diagram about our study and further exploration in Figure 6 according to the relative literature. Although the current study had not clearly explained the mechanism of hypoxia and the relationship between DQYS and hypoxia, it at least provided some information and constructions for our hypothesis and the further study. 

In conclusion, our findings suggested that chronic intermittent hypoxia could induce similar symptoms and signs accorded with the clinical features of DQYS in mice, and simulate the pathogenesis characteristic from deficiency to blood stasis. These results would be helpful to establish a suitable animal model for reasonably evaluating Chinese prescriptions for the treatment of this syndrome, exploring pathological mechanism of the syndrome, clarifying the relationships between the syndrome and related diseases and facilitating the communication between TCM and modern medicine. Further investigations are underway to clarify the precise pathogenesis why chronic intermittent hypoxia induced these results.

##  Funding

National Nature Science Fund (30772792).
